# Combined Cocaine Hydrolase Gene Transfer and Anti-Cocaine Vaccine Synergistically Block Cocaine-Induced Locomotion

**DOI:** 10.1371/journal.pone.0043536

**Published:** 2012-08-17

**Authors:** Marilyn E. Carroll, Natalie E. Zlebnik, Justin J. Anker, Thomas R. Kosten, Frank M. Orson, Xiaoyun Shen, Berma Kinsey, Robin J. Parks, Yang Gao, Stephen Brimijoin

**Affiliations:** 1 Department of Psychiatry, University of Minnesota, Minneapolis, Minnesota, United States of America; 2 Departments of Medicine, Psychiatry & Neuroscience, and Pathology & Immunology, Baylor College of Medicine and Veterans Administration Medical Center, Houston, Texas, United States of America; 3 Regenerative Medicine Program, Ottawa Hospital Research Institute, Ottawa, Ontario Canada; 4 Department of Molecular Pharmacology and Experimental Therapeutics, Mayo Clinic, Rochester, Minnesota, United States of America; California Pacific Medicial Center Research Institute, United States of America

## Abstract

Mice and rats were tested for reduced sensitivity to cocaine-induced hyper-locomotion after pretreatment with anti-cocaine antibody or cocaine hydrolase (CocH) derived from human butyrylcholinesterase (BChE). In Balb/c mice, direct i.p. injection of CocH protein (1 mg/kg) had no effect on spontaneous locomotion, but it suppressed responses to i.p. cocaine up to 80 mg/kg. When CocH was injected i.p. along with a murine cocaine antiserum that also did not affect spontaneous locomotion, there was no response to any cocaine dose. This suppression of locomotor activity required active enzyme, as it was lost after pretreatment with iso-OMPA, a selective BChE inhibitor. Comparable results were obtained in rats that developed high levels of CocH by gene transfer with helper-dependent adenoviral vector, and/or high levels of anti-cocaine antibody by vaccination with norcocaine hapten conjugated to keyhole limpet hemocyanin (KLH). After these treatments, rats were subjected to a locomotor sensitization paradigm involving a “training phase" with an initial i.p. saline injection on day 1 followed by 8 days of repeated cocaine injections (10 mg/kg, i.p.). A 15-day rest period then ensued, followed by a final “challenge" cocaine injection. As in mice, the individual treatment interventions reduced cocaine-stimulated hyperactivity to a modest extent, while combined treatment produced a greater reduction during all phases of testing compared to control rats (with only saline pretreatment). Overall, the present results strongly support the view that anti-cocaine vaccine and cocaine hydrolase vector treatments together provide enhanced protection against the stimulatory actions of cocaine in rodents. A similar combination therapy in human cocaine users might provide a robust therapy to help maintain abstinence.

## Introduction

Recent animal and human studies have yielded promising results with antibody- and enzyme-based approaches to treatment of cocaine addiction by intercepting drug molecules before they reach the brain. This line of work began with investigations of anti-cocaine antibodies [1], [2], later including monoclonal antibodies [3], [4], and vaccines [5], [6], [7]. Efforts then expanded with studies of cocaine metabolizing enzymes based on human plasma butyrylcholinesterase (BChE) [8], [9], [10], [11], or bacterial cocaine esterase [11], [12], [13].

Each type of interception agent has merits. Antibodies can be expected to be safe, are effective against moderate doses of cocaine in some animal models [4], and are long lasting when generated by vaccination [14], [15]. Recently, in its first clinical trial, an anti-cocaine vaccine showed modestly positive results, including an ability to increase the frequency of drug-free urine samples in a population of street cocaine users [16]. Meanwhile, BChE-based cocaine hydrolases such as CocH, a quadruple mutant developed with the aid of site-directed mutagenesis [17], [18], have acquired a catalytic efficiency that drastically accelerates cocaine elimination in rats and mice [19], [20], [21]. By employing novel gene-transfer vectors, particularly helper-dependent adenoviral vectors, it has been possible to generate high circulating levels of CocH or similar enzymes in rats and mice for a year or more after a single treatment [22]. Following a previous history of self-administration these levels of expression prevented renewed drug-seeking for at least 6 months when rats in forced abstinence were challenged with i.p. cocaine [23]. The effective blockade of this “drug-primed reinstatement behavior", an animal model of relapse, supports the concept that drug interception in the peripheral circulation may aid recovering drug users to avoid resumption of drug-taking.

It remains uncertain whether either enzyme or cocaine antibody alone can be sustained at clinically useful levels throughout the initial months of abstinence when risk of relapse is highest. For that reason we considered combining these two therapeutic approaches for enhanced effectiveness. Setting the stage, we found that, even when cocaine is bound to antibodies with nanomolar affinity, it is hydrolyzed by CocH almost as quickly as when it is free [24]. Such results suggested that antibody and enzyme in combination could provide both rapid binding and greatly accelerated metabolism of incoming cocaine in a fashion not readily surmountable by repeated large doses. In the present study cocaine-induced locomotor activity and cocaine-induced locomotor sensitization were used as robust behavioral endpoints to explore this hypothesis in mice and rats treated with antibody (delivered directly or by vaccination) and/or enzyme (delivered directly or by vector-mediated gene transfer). Although sensitization and drug reward can be dissociated [25], animal studies indicate that these behaviors are related to the development of drug addiction [26], [27]. Therefore, we considered drug-induced locomotion and sensitization of locomotor activity as useful predictors of treatment effects on ultimate drug dependence.

In the following studies we first established mouse and rat models in which the efficacy of cocaine hydrolase and anti-cocaine antibodies could be compared, alone and in combination, with regard to preventing central actions of cocaine. Experiments tested the following hypotheses: 1) the presence of anti-cocaine antibodies will modestly reduce cocaine-stimulated locomotion and sensitization; 2) the presence of cocaine hydrolase will have a similar effect; 3) the combined presence of cocaine antibodies and hydrolase will reduce cocaine-driven behavior further than either treatment alone.

## Results

### Mouse experiments

**Figure 1:**
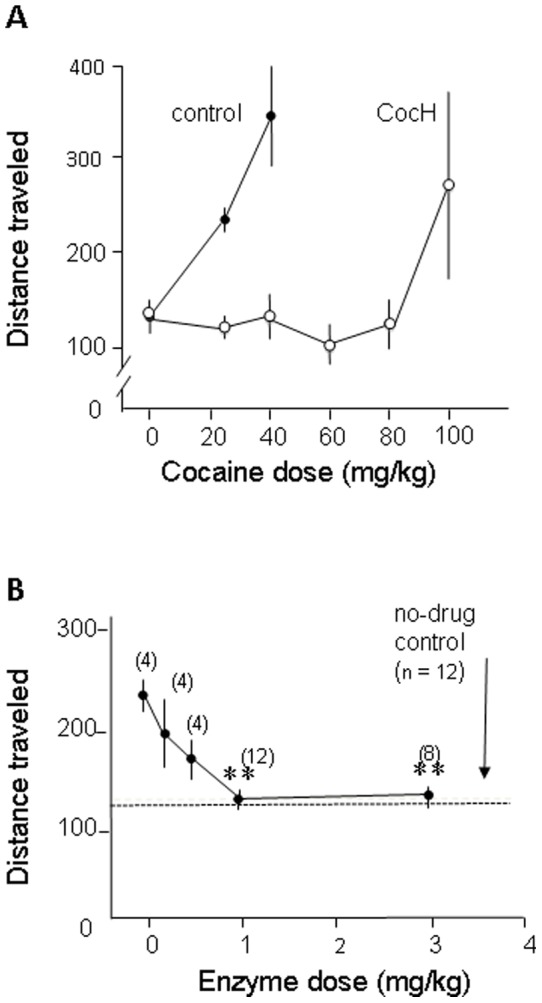
CocH suppresses locomotor response to cocaine in mice. **A.** Distance traveled (average cm per 2 min bin) as a function of cocaine dose in the presence and absence (control) of cocaine hydrolase (CocH, 1 mg/kg, i.p). All groups, n = 12 (means ± SEM). **B**. Distance traveled after a fixed dose of cocaine (40 mg/kg i.p.) as function of enzyme dose (mg/kg). Group sizes, N = 4 to12 (numbers in parenthesis).

Initial studies were carried out to determine the optimal cocaine dose for producing locomotor stimulation in mice and for antagonizing that stimulation with enzyme delivered by i.p. injection. In otherwise untreated animals, cocaine at 40 mg/kg reliably evoked a highly significant 3-fold increase in distance traveled during a 1-hr period of observation (Fig. 1A). Enzyme pretreatment (1 mg/kg i.p., 2 hr before testing) caused a ∼ 5-fold rightward shift in the dose response curve for cocaine stimulation. When increasing doses of hydrolase were tested against a fixed 40-mg/kg dose of cocaine, drug-induced locomotion fell progressively, with complete suppression by enzyme injections at 1 mg/kg or above (Fig. 1B). CocH by itself (i.e., without cocaine treatment) did not affect spontaneous locomotion (data not shown).

**Figure 2:**
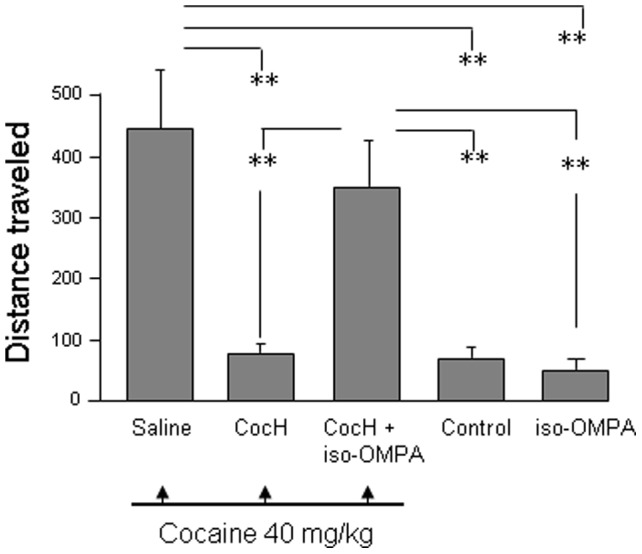
Selective enzyme inhibitor relieves CocH suppression of locomotor response. Mice (groups of 8) habituated to locomotor activity chambers were given enzyme (CocH, 1 mg/kg, i.p.) or inhibitor (iso-OMPA, 50 mg/kg, s.c.) or both, followed 3 hr later by cocaine (40 mg/kg, i.p.). Controls received either i.p. saline or iso-OMPA alone. ** p<0.01.

To discover whether CocH suppresses locomotor effects entirely by selective enzymatic destruction of cocaine, similar experiments were conducted in mice treated with iso-OMPA, an irreversible organophosphate anticholinesterase selective for BChE and BChE-mutants like CocH (Fig. 2). Two hr before cocaine exposure, this inhibitor was given s.c. at 50 mg/kg (a high dose that proved necessary for >95% inactivation of CocH in this species). Enzyme was administered i.p at the same time (see Methods). When tested 2 hr later, the locomotor effect of cocaine (40 mg/kg i.p.) was virtually as strong in these mice as in animals pretreated only with saline. In contrast, locomotor stimulation was again absent in mice pretreated with 1 mg/kg CocH without iso-OMPA. A control experiment showed that iso-OMPA on its own had no stimulatory action. In fact, it caused either no effect or a small decrease in spontaneous locomotion (Fig. 2).

Next, we examined the hypothesis that anti-cocaine antibody (see Methods) would also protect against cocaine’s stimulating effects on locomotor behavior, by itself, and would add to the protection when used in combination with enzyme. Since a small dose of CocH already prevented stimulation by cocaine at 40 mg/kg (Fig. 1), a more challenging test was performed with a much higher dose of cocaine (120 mg/kg i.p.). Preliminary work indicated that this dose was lethal as a single bolus, but all animals survived when it was given as two 60-mg/kg injections spaced 10 min apart. To generate circulating anti-cocaine antibody for the test, one group of mice was vaccinated with carrier-conjugated cocaine hapten approximately 5 weeks beforehand (see Methods). Plasma antibody levels during the testing week were all above 220 µg/ml and averaged 550±80 µg/ml. Other mice were given a direct injection of serum from vaccinated donor mice (16 mg/kg of specific anti-cocaine IgG, i.p., 2 hr before testing), which led to circulating antibody levels of 25 to 50 µg/ml.

**Figure 3:**
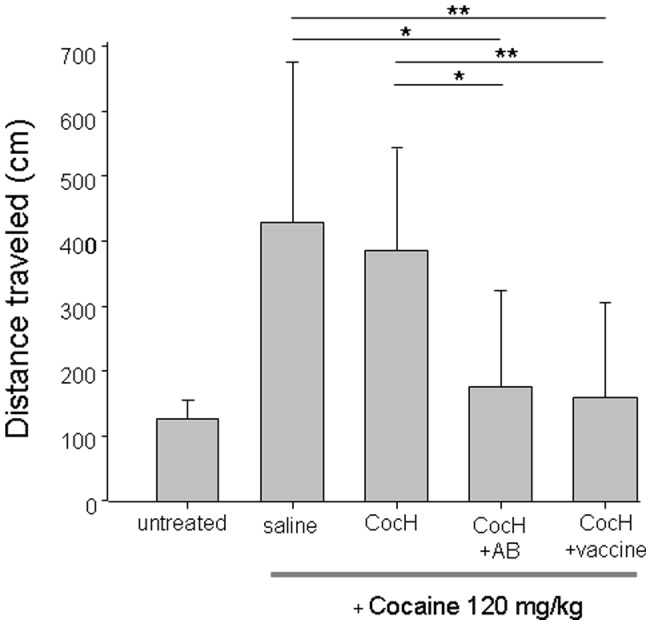
Combined anti-cocaine antibody and cocaine hydrolase block mouse locomotor stimulation by high-dose cocaine. Untreated mice were given only i.p. saline. All others received cocaine, total of 120 mg/kg i.p., in two 60 mg/kg doses 10 min apart. CocH groups received enzyme, 1 mg/kg, i.p. AB mice received anti-cocaine antibody, 16 mg/kg i.p. “Vaccine" mice were each immunized with 100 µg of norcocaine-conjugate cocaine vaccine, and a booster immunization at 3 weeks. Statistical significance (n = 8 for all groups): * p<0.05; ** p<0.01).

In the locomotor tests, mice with only saline pretreatment showed a large cocaine-induced increase in distance travelled (Fig. 3). Mice given CocH (1 mg/kg i.p.) exhibited a nearly equal cocaine effect (i.e., only marginal enzyme protection) as expected. However, mice given CocH along with anti-cocaine antibody injection showed a greatly reduced locomotor effect from cocaine, exhibited no other behavioral signs, and outwardly resembled mice not exposed to any stimulant. The same result was seen in mice given CocH after anti-cocaine vaccination. In contrast, mice pretreated *only* with antibody injection or vaccination showed signs of profound weakness and remained lying on the chamber floor for most of the observation period (data from these groups with essentially zero locomotion are not shown).

### Rat experiments

**Figure 4:**
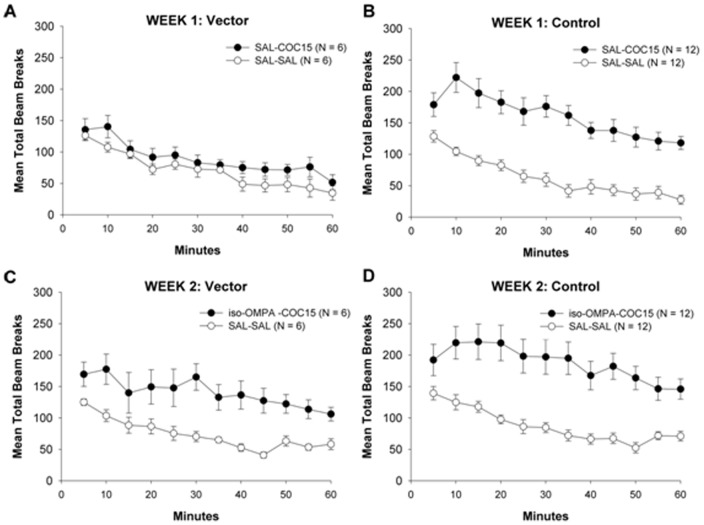
CocH suppresses *rat* cocaine locomotor response but enzyme inhibitor relieves suppression. Rat locomotor activity is shown in “beam breaks" (mean ± SEM) per 5 min bin over a 60-min period of observation following an i.p. test injection of cocaine (15 mg/kg) or saline. On week 1 (**A** & **B**) rats were pretreated with i.p. saline, 2 hr before cocaine injection. On week 2 (**C** & **D**) they were pretreated at the corresponding time with i.p. iso-OMPA (1.5 mg/kg). “Vector" rats (N = 6) received 10^11^ particles of helper-dependent adenovirus via the tail vein approximately 1 month before testing, and a satisfactory level of cocaine hydrolase activity (>150 mU/ml) was confirmed in plasma samples drawn immediately after the final experiment. Control rats (N = 12) were pretreated only with saline. ANOVA confirmed a significant effect of cocaine injection in all groups except vector-treated rats in the absence of iso-OMPA.

In order to confirm that effects of CocH vector in rats also depended on enzyme activity, experiments were again performed with the selective inhibitor, iso-OMPA. Because rats are more sensitive than mice to iso-OMPA, much less inhibitor was needed: 1.5-mg/kg, i.p., eliminated 99% of the plasma cocaine hydrolase activity but less than 5% of the acetylcholinesterase activity. As with mice, control rats showed a robust locomotor response to i.p. cocaine, 15 mg/kg, in comparison to i.p. saline (Fig. 4B), while CocH-expressing vector-pretreated rats showed almost no cocaine response (Fig. 4A). When the same animals were re-tested one week later, 2 hr after iso-OMPA, the vector-treated rats showed a highly significant locomotor response to cocaine injection as compared with saline (Fig. 4C). But in the group without vector, iso-OMPA did not change the cocaine response significantly from a week earlier (Fig. 4D).

Cocaine-induced locomotor activity was assessed further with a cocaine-sensitization procedure modeled after Ferrario et al. [28]. This procedure utilized four experimental conditions in the following order: 1) 3 days of acclimation, 2) 8 days of cocaine training, 3) 15 days of drug-free “rest", and 4) a final “sensitization challenge" with cocaine (see Table 1 for outline and Methods for details). Cocaine doses in these experiments were uniformly 10 mg/kg. The following groups of rats were studied: 1) VAC (pretreated with vaccine); 2) VEC (pretreated with CocH vector); 3) VAC+VEC (pretreated with both agents); and 4) Control (pretreated only with saline).

**Table 1:** Scheme for testing locomotor responses to cocaine.

	Acclimation	Training	No Drug	Challenge
Day	1–3	4–12	13–28	29–30
Treatment	N/A	S C C C C C C C C	N/A	S C
Duration	3 days	9 days	15 days	2 days
Locomotor	Yes	Yes	No	Yes
Session Length (min)	45	90	N/A	90

Rat locomotor experiments were carried out in four distinct phases. Phase 1, “Acclimation", consisted of 3 consecutive days of 45-min in the test chamber with no interventions. Phase 2, “Training", involved 9 days of 90 min in the chamber with an i.p. injection of saline (S) or cocaine, 10 mg/kg C) at the 45-min time point. Phase 3, “No Drug", involved 15 “rest days" in the home cage with no interventions. Phase 4, “Challenge", was 2 days in the chamber with i.p. saline or cocaine at the respective 45-min time points.

Table 2 shows mean plasma concentrations of antibody (µg/ml) and enzyme (mU/ml) at study onset (beginning of training phase) and end (day of cocaine challenge). These concentrations were in line with previous results in our laboratories [22], [29]. In particular, there was no sign that vaccination affected enzyme transduction by vector, or vice versa. Equally important, the measured values did not differ significantly during the two testing periods for any group, indicating that protein levels were in general sustained throughout the procedure.

**Table 2:** Average antibody (µg/ml) and/or enzyme (mU/ml) levels for the VAC, VEC, VEC+VAC treated rats during the Training and Cocaine Challenge Phases.

Groups	Training Phase		Challenge Phase	
	*Antibody* *(*µ*g/ml)*	*CocH* *(mU/ml)*	*Antibody* *(*µ*g/ml)*	*CocH* *(mU/ml)*
*VAC*	1080±382		1501±199	
*VEC*		1310±442		760±270
*VEC+VAC*	1587±268	1540±396	1813±182	1123±338

Cocaine hydrolase activity and anti-cocaine antibody levels were assessed midway through the training phase (day 8) and at the onset of the challenge phase (day 29). Means and standard errors are shown. Neither of the measured variables changed significantly across the course of the experiment, and there were no significant differences for either variable across treatment groups.

No significant group differences in locomotor activity emerged during the three 45-min acclimation sessions before the training phase (data not shown). Immediately after each 45 min habituation period, during the training phase, rats received an i.p. injection of saline (training day 1) or cocaine (training days 2–9) and locomotor activity was assessed for an additional 45 min. ANOVA results indicated a significant main effect of treatment group (F_3, 169_  = 3.93, p<0.05) and injection condition (F_4, 169_  = 11.06, p<0.01), but there was no significant group-by-treatment interaction in locomotor activity during the habituation periods that preceded injection.

**Figure 5:**
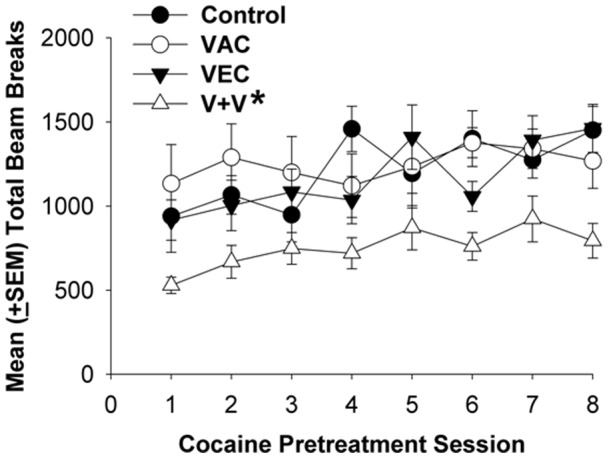
Mean daily beam breaks after cocaine during training phase. Data represent rat locomotor activity in 8 daily 45 min sessions immediately following cocaine injections. Statistical significance: * significantly *lower* locomotor activity in VEC+VAC group compared to all other groups (p<0.05); **†** significantly *greater* locomotor activity on the final training-phase day compared to the first day (p<0.05).

Across the cocaine-training period, during the 8 daily 45-min sessions preceded by cocaine injections, there was a general increase in total daily cocaine-induced locomotor responses (Fig. 5). Over this period there was a main effect of treatment group (F_3, 271_ = 5.22, p<0.01) and day (F_7, 271_ = 4.90, p<0.0001) but no significant group-by-day interaction. Post hoc comparisons revealed significantly greater cocaine-induced locomotor activity on the final training session compared to the first day (p<0.05), and lower activity in the VEC+VAC group compared to all others (p<0.05). These results established that repeated cocaine injections over 8 days produced sensitization of locomotor activity, but with a much reduced effect in the VEC+VAC group.

**Figure 6:**
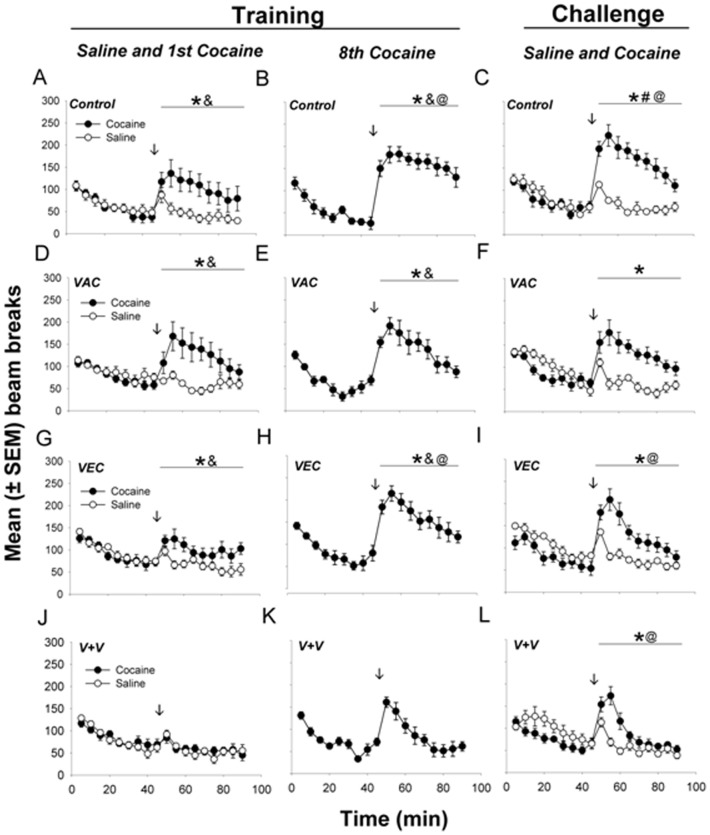
Combined vaccine and vector treatment reduces cocaine-induced locomotor activity even after repeated cocaine injections. Shown are beam breaks per 5 min interval (mean ± SEM). Left panels: initial two days of training phase. Middle panels: last day of training phase. Right panels: both days of challenge phase. Statistical significance: * significantly higher locomotor activity after cocaine injections compared to saline (p<0.05); **&** significantly higher cocaine-induced locomotor activity than in the VAC+VEC group (p<0.01); **@** significantly higher cocaine-induced locomotor activity after the last cocaine injection compared to first injection (p<0.01); **#** significantly greater cocaine-induced locomotor activity in control group compared to all others (p<0.05).

All rats (regardless of group status) exhibited a low level of locomotor activity after saline injections. Thus, the procedure of i.p. injection, *per se*, had a minimal effect on locomotion. By contrast, cocaine induced a locomotor response that significantly exceeded the response to the preceding saline injection (p<0.01) in all groups but one (VAC+VEC). There was also a significant main effect of treatment (F_3, 169_  = 3.63, p<0.05) and injection (saline or cocaine; F_4, 169_  = 46.11, p<0.01) and a significant treatment-by-injection interaction (F_12, 169_  = 2.73, p<0.05). Post-hoc analyses based on the entire 45-min period of observation following cocaine injection indicated sensitization of drug-induced locomotor activity in the Control (Fig. 6A and 6B) and VEC groups (Fig. 6G and 6H). This sensitization was indicated by a significant increase (p<0.01) in locomotor activity when the 8^th^ day of cocaine injections was compared to the first day. The same analysis showed no sensitization in the VAC or VAC+VEC groups (Fig. 6F and 6L). The double-treated group (VAC+VEC) was exceptional. These rats exhibited no locomotor response to the initial cocaine treatment. In addition, between-group comparisons indicated that locomotor activity in the VAC+VEC group after the last cocaine-training injection was also lower (p<0.01) than in all other groups (Fig. 6J and 6K).

Behavioral sensitization to cocaine persisted throughout the 15-day drug-free period between training and challenge phases. Thus, no group showed a significant difference between the 8^th^ day of cocaine training and the cocaine challenge day. In other words the responses to cocaine on the challenge day were all maintained at the level reached after the last (8^th^) cocaine injection in the training phase, 15 days earlier. The control rats showed more locomotor activity on the challenge day than any other group (p<0.05, Fig. 6C), but all except the VAC rats responded to cocaine challenge with significantly greater locomotor activity (p<0.05) than after the first cocaine training injection (compare left and right hand panels of Fig. 6).

The relationship between cocaine-induced locomotor activity and blood levels of antibody and enzyme was tested by correlation analysis for the training and challenge conditions. Negative correlations were anticipated, but, of the 8 separate comparisons (2 measures, 2 treatment groups, 2 conditions), the only statistically significant results to emerge were with regard to CocH levels in the vector-treated rats during the cocaine challenge. These were in opposite directions for the VEC group (r = −0.76, p<0.01) and the VEC+VAC group (r = 0.73, p<0.05). Thus, it appeared that treatment effects did not vary strongly or predictably with variations in antibody and enzyme levels over the range exhibited by the animals.

## Discussion

The present study confirms past animal work indicating that anti-cocaine antibodies or efficient cocaine-hydrolyzing enzyme can reduce behavioral responses to cocaine [3], [23], [30], [31]. This type of protective effect was apparent in tests run independently in our two laboratories on two animal species (rats at University of Minnesota, mice at Mayo Clinic). In both series of experiments, tests showed that the effects of CocH treatment, whether by protein injection or by viral gene transfer, depended absolutely on the destruction of cocaine by active enzyme. Thus, control-level locomotor responses to cocaine reappeared in animals treated with the enzyme inhibitor, iso-OMPA, which had no stimulatory action on its own. We conclude that CocH suppresses cocaine-induced activity in mice specifically by hydrolyzing the stimulant.

An equally clear and still more interesting outcome was the enhancement of protection when CocH was combined with anti-cocaine antibody delivered either directly or by vaccination. Thus, under certain circumstances, levels of cocaine antibody and CocH that reduced cocaine-induced locomotor stimulation only moderately, when present alone, suppressed responding completely when present together. That outcome is consistent with the view that the two agents act to reduce cocaine’s rewarding effects in a complementary, or even synergistic manner. Additional experiments are needed for a rigorous test of synergism, with more animals, doses, and isobolographic analysis [32]. Nonetheless, these data establish that CocH and anti-cocaine antibodies have at least additive effects. Furthermore, our finding that gene transfer and immunization can occur together without weakening enzyme transduction or antibody response suggests that such combined treatments should be feasible in clinical practice.

The present interventions had mutually reinforcing actions on cocaine-stimulated behavior in both mice and rats. Under the conditions examined here, mice were at best partly protected by single treatment with antibody, vaccine, or enzyme. An adverse effect of high dose cocaine, namely weakness or prostration, was even greater in the animals pretreated only by antibody or cocaine vaccine than in saline-treated controls. This effect may be novel and we have no simple explanation. It might have reflected actions in the periphery, intensified by enhanced cocaine blood levels owing to antibody binding. However, that hypothesis is difficult to reconcile with the basic assumption that antibody-bound drug should be unavailable to target proteins. Alternatively, one could propose that antibody served to prolong cocaine’s residence time in the circulation and thereby shifted the balance between central and peripheral effects. In any case this is a matter that deserves further experimental study.

In striking contrast, mice protected with both antibody and enzyme showed little locomotor response to a massive cocaine dose and remained “phenotypically normal", with no sign of motor weakness or stereotypy. At the least, this outcome demonstrates that these two treatment modalities combine efficiently in terms of therapeutic outcome, and might exert synergy.

In rats as in mice, locomotor stimulation during initial “cocaine training", was almost unaffected by vaccine alone, and was only partially reduced by vector treatment alone, despite the substantial levels of anti-cocaine antibody and CocH, respectively. This result allowed further probing of the efficacy of combined treatment, which was confirmed by near-total elimination of cocaine-stimulated locomotor responses in the VAC+VEC group during the training phase.

The effect of these same treatments on sensitization (higher responding to cocaine on the last versus the first day of training) was more complex. Both the controls and the vector-only rats exhibited clear sensitization. On the other hand, the doubly treated rats did *not* show significantly greater responding across the same days. This outcome suggests that sensitization was impaired when antibody and enzyme were both present, but the data do not allow us to conclude it was abolished. Another measure of sensitization can be taken from the increased responding to cocaine on the Challenge Day (after a 15-day break from cocaine injections) as compared with the first day of cocaine exposure. This measure indicated significant sensitization in all except the VAC-only rats, and there again, the data do not prove it was absent in that group. In interpreting these results it is well to bear in mind recent findings using intracranial stimulation, which have dissociated the effects of repeated cocaine on motor processes and reward [33]. Thus, while sensitization to the locomotor effects of cocaine may model some aspects of human addiction, changes in cocaine-induced locomotion may occur independently of changes in reward perception. Therefore, conclusions about reward processing among the treatment groups must be tempered.

Overall, however, the results of the present study show promising effects of vaccination and cocaine hydrolase gene transfer, especially in combination, as treatments to modify cocaine-induced behavior. Even though cocaine-stimulated locomotor behavior is not necessarily a reward-driven phenomenon, it is certainly relevant to drug addiction [26], [27]. Therefore, the strong and possibly synergistic reduction of cocaine-stimulated locomotion in rats and mice suggests that combination of cocaine antibody and cocaine hydrolase would also provide a robust reduction of reward-driven behavior compared with single treatments with either agent. It will be important to confirm the additive effects of vaccination and hydrolase transduction in models of drug-seeking behavior, including tests of drug-primed reinstatement and ongoing responding for cocaine reward.

In our view, combined treatments with enzyme and antibody should be explored further with two potential aims in view. First, if dual treatments succeed in generating high levels of cocaine antibody and cocaine hydrolase in human users, they are likely to impose a substantial barrier to the resumption of drug-seeking behavior–one that would be difficult to surmount, even with large or repeated doses of cocaine. Thus, a goal would be to compare the efficacy of single and combined treatments in experimental paradigms that model the escalation of cocaine self-administration during periods of extended access. Secondly, if our findings in rats and mice translate to human cocaine users, dual treatment would provide a degree of therapeutic protection to those who are poor responders to the vaccine, or to the vector, or to both modalities. Poor responding to anti-drug vaccines has presented a serious obstacle in clinical trials both with cocaine users and tobacco users [34], [35] and an adjunctive therapy could have major impact.

Another important advantage to dual treatment is the potential for improved safety. Like most other vaccines, anti-cocaine vaccines appear to present few if any risks to the recipient [14], [15], [36]. At the current stage of development, however, there are legitimate concerns about the risk of viral vectors. Studies have shown that these risks primarily arise during the initial stages of vector delivery, as the modified virus circulates on its way to insertion in target host cells [37], [38], [39]. In this phase, innate immune reactions that have evolved to protect against viral infection can generate high levels of interleukins and other cytokines that adversely impact host physiology [40], [41]. In addition, cytotoxic T-lymphocytes may be stimulated to kill host cells in the process of incorporating a vector, a dangerous and potentially life threatening outcome with hepatotropic vectors, including conventional and helper-dependent adenoviral vectors as well as adeno-associated viral vectors [42]. Vector-related toxicity, however, is strongly dose-dependent. Therefore, it is an absolute requirement that vector doses must be well below the levels ever associated with serious adverse effects in experimental animals, including non-human primates, or human subjects. If single treatment with vector were the only option, its margin of safety might preclude levels of transgene expression that are sufficient for the desired impact. On the other hand, if modest levels of expressed hydrolase are supra-additive with anti-cocaine antibodies, it should be possible to obtain real therapeutic effects from vector levels that are entirely safe.

Further enhancing the prospects for safe and effective hydrolase treatment is a body of compelling evidence that native BChE, from which cocaine hydrolases derive, is essentially devoid of toxicity and, for that matter, detectable physiologic actions of any sort. Thus, in studies designed to evaluate BChE for protection against chemical warfare agents, Saxena and co-workers [43], [44] found no clinical signs in mice and guinea pigs given enzyme doses that raised plasma levels 50- to 100-fold. A later study with guinea pigs and rats used BChE doses up to 0.5 g [45]. Although plasma BChE rose nearly 500-fold above levels typical for human adults, detailed evaluation revealed no sign of altered blood pressure, thrombogenesis, or pulmonary inflation pressure, and no *postmortem* tissue pathology. Finally, unpublished human studies conducted by the Dept. of Defense failed to observe physiological effects from gram quantities of the same enzyme (D. Cerasoli, USAMRICD, personal communication).

Continued BChE mutagenesis should further enhance the safety and efficacy of hydrolase gene transfer. The CocH used here is highly efficient in cocaine hydrolysis, but Zhan and others have recently identified other BChE variants with even greater catalytic efficiency [21], [46], [47]. In our hands these new mutants also transduce readily and are stable in vivo (Brimijoin, Gao, preliminary results), pointing the way to greater therapeutic suppression of cocaine effects with smaller doses of vector and enzyme.

Significant improvement in vaccine performance is also desirable and should be possible. Unfortunately, one of the most effective cocaine vaccines reported to date relies on capsid proteins of disrupted adenovirus [48], which are likely to generate immune responses that prevent gene transfer with any of the vectors we have explored. But other carrier proteins and adjuvants may well prove superior to those employed so far [49], [50]. Finally, additive benefits could well be achieved by combining further modalities, including behavioral therapy. That sort of multipronged strategy may in fact be ideally suited to a multifaceted illness like cocaine addiction.

## Materials and Methods

### Animal Subjects

#### Ethics Statement

The experimental protocols for rats (1008A87756) and mice (A26810) were approved, respectively, by the University of Minnesota and Mayo Clinic Institutional Care and Use Committees. All experiments were conducted in accordance with the Guide for Care and Use of Laboratory animals [51] in laboratories accredited by the American Association for the Accreditation of Laboratory Animal Care.

#### Mice

A total of 244 Balb/c male mice were obtained at 6 to 7 weeks of age from Harlan Sprague Dawley (Madison WI). Sixteen mice received anti-cocaine vaccine consisting of a norcocaine adduct conjugated to keyhole limpet hemocyanin (8100-1 KLH SNC), 5.7 mg/kg, 100 µg/mouse, injected i.m. into the upper thigh of each hind leg in a volume of 80 µl per site. After three weeks a booster immunization was given in the same dose. At four weeks, a small blood sample was taken from each mouse to determine the levels of specific anti-cocaine antibodies (see below).

Proteins and drugs were administered to mice by more than one route, but i.p. was favored for most purposes (injection volumes were 6.8 µl/g). In our experience (Anker et al., 2012), even sizeable proteins like IgG (150 kDa) and CocH dimers (170 kDa) are absorbed from the peritoneal space. When these proteins are given i.p., plasma levels 2-hr or more later approach those measured after same-dose tail vein injections, indicating similar bioavailability and distribution. The i.p. route also permitted rapid treatment of multiple mice and eliminated stress from restraint needed for i.v. injection. Finally, we established that a small volume of i.p. CocH on the left side does not interact substantially with a small volume of i.p. cocaine administered on the right side 1 hr or 2 later. However, in the one experiment with *three* different agents, iso-OMPA was given s.c. to ensure that it would solely inhibit pre-absorbed enzyme.

#### Rats

Fifty-two experimentally naïve 90-day old male Wistar rats weighing 350 to 400 g (Harlan-Sprague Dawley, Madison, WI) were used in total. Locomotor studies with the selective enzyme inhibitor, iso-OMPA, used 6 vector-treated rats (10^11^ viral particles via tail vein injection approximately 1 month before testing) and 12 vector-naïve rats. On week 1, rats received i.p. cocaine (15-mg/kg) 2 hr after pretreatment with i.p saline, and locomotor activity was recorded for 60 min. On week 2, rats received the same dose of cocaine 2 hr after pretreatment with i.p. iso-OMPA (1.5 mg/kg), and locomotor activity was recorded again. For studies of cocaine-induced locomotor activity in more depth, including measures of sensitization, another set of age- and weight-matched rats were allocated to the four treatment groups in the following numbers: VAC (n = 8), VEC (n = 10), VEC+VAC (n = 8), and untreated controls (n = 8). Vaccine (250 µg per rat, i.p.) and/or vector (10^11^ viral particles via tail-vein) were delivered approximately five weeks prior to locomotor testing to allow for the production of high antibody and enzyme levels. For dual treatments, approximately half the rats received vector first and were then vaccinated four weeks later (to avoid potential immunologic reactions to vector before it inserted into liver cells). The other rats received vector and the initial vaccination on the same day. Since the peak levels of enzyme expression were equivalent after both procedures, the two groups were combined for further study.

Before locomotor testing, and after each experimental session, rats were housed individually in plastic cages with free access to water and food (Purina Laboratory Chow, Purina Mills, Minneapolis, MN, USA) in rooms controlled for temperature (24°C), humidity (40–50%), and light (light/dark, 12hr/12-hr with lights on at 6:00 a.m.).

### Mouse Locomotor Apparatus and Procedure

Cocaine-induced locomotion in mice was assessed in sound-insulated rectangular activity chambers from Med Associates Inc, St Albans, VT USA (27-cm W×27-cm L×20-cm D) with continually running fans and infrared lasers and sensors. Beam breaks were assessed in 2-min bins over 60 min, converted automatically to distance travelled (cm) and recorded on a computer with Med-PC software Version 4.0.

Untreated mice and mice immunized 5 weeks earlier with KLH-norcocaine were habituated by the following procedure. Before entering the locomotor chamber (time zero) each animal received i.p. saline (6.7 ml/kg). A second saline injection was given at 60 min, and locomotor activity was recorded for 1 hr. After three days of habituation, acute pretreatments were administered in a series of similarly timed i.p. injections: 1) at time zero, saline or enzyme (CocH, 1 mg/kg, i.p.) and/or anti-cocaine antiserum (AB, 16 mg/kg, i.p.); and 2); at 60 min, cocaine hydrochloride (120 mg/kg, in two 60-mg/kg doses spaced 10 min apart). The habituation and control procedures were similarly modified, with equivalently spaced injections of saline in place of cocaine.

### Rat Locomotor Apparatus and Procedure

A circular activity monitor with an inner diameter of 35.6 cm and an outer diameter of 59.6 cm was used to measure locomotor activity (Model ENV-580, Med Associates Inc). The apparatus was equipped with 4 infrared sensors positioned 5 cm above the floor at 0°, 90°, 180°, and 270°. An activity response was counted each time a photo beam sensor was interrupted, but when two or more beam breaks occurred sequentially at the same photo beam they were counted as one. Sensor beam breaks were measured in 5 min bins and recorded with Med-PC software (Version 4.0, Med Associates) installed on PC computers. Owing to the limited spatial resolution, the primary data were not converted into linear distances.

Locomotor sessions were conducted once daily during the light phase of the light/dark cycle according to a procedure modified from Li et al. [52] and outlined in Table 1. This procedure was selected, as it reliably induces locomotor sensitization in rats and has been sensitive to pharmacological treatment effects [28]. Initially, locomotor activity was assessed during 3, 45-min habituation sessions to allow rats to acclimate to the apparatus and to determine potential baseline differences in locomotor activity. Following the 3-day acclimation condition, the training phase commenced. Each training session began with a 45-min habituation period to establish a baseline and to control for the effects of stress of injection on locomotor activity, and on the 8 remaining days, a 10 mg/kg i.p. cocaine injection was administered. After the final training day, rats were placed back in their respective home cage and locomotor testing and injections were discontinued for 15 days. Subsequently, locomotor activity was reassessed following a single saline or cocaine i.p. injection according the methods described for the training phase (Table 2).

### Blood Collection

Small blood samples (<0.1 ml) were taken from mice by cheek puncture using a 21-gauge mouse-bleeding lancet. A sterile gauze pad was then applied with slight compression for less than a minute to stop the bleeding. Rats to be sampled were briefly restrained in a Plexiglas tube and the free end of the tail was submerged in warm water to increase visibility of the lateral tail vein. A 23-gauge butterfly infusion needle was inserted into the vein, approximately one-third the distance from the tail tip, and gently suctioned until ∼0.2 ml of blood was obtained. All blood samples were centrifuged for 15 min in serum separator tubes (Becton Dickenson, Franklin Lakes, NJ USA) and stored at −20°C before being analyzed for antibody and active CocH enzyme levels.

### Calibration of anti-cocaine antisera

Aliquots of antibody-rich mouse plasma drawn according to IACUC guidelines were tested routinely for anti-cocaine IgG levels. Each sample was treated with diisopropylfluorophosphate (10^−5^ M) for 5 min (to inactivate cocaine hydrolysis by blood-borne enzymes) and then incubated with ^3^H-cocaine in near saturating concentration (5 µM). After 50 min, a 50-µl aliquot of treated sample was transferred to a Centricon Sepharose spin-column for centrifugation at 1000×g for 4 min, and 30 µl of the void volume fraction was mixed for scintillation counting with 0.1 M sodium carbonate and 4 ml “BioSafe" fluor (RPI Inc, Mt Prospect IL USA). Control experiments showed that >80% of the sample protein passed into the collection tube (including IgG with bound cocaine), but <2% of free ^3^H-cocaine. The assay signal (counts per min) was linearly proportional to IgG concentration over a wide range and was calibrated with reference to a standard of purified anti-cocaine IgG run along with test samples.

### Enzyme and drug sources

The BChE-based cocaine hydrolase, CocH, was prepared from clonal lines of stably transfected Chinese hamster ovary cells by purification on DEAE Sepharose followed by ion exchange chromatography as previously described [53] and was stored at −80°C until time of use. Cocaine HCl was obtained from the National Institute of Drug Abuse (Research Triangle Institute, Research Triangle Park, NC USA). This drug was freshly dissolved in 0.9% NaCl for each mouse experiment at a concentration that allowed delivery of 200 µl per 30 g (typical subject weight).

### Enzyme assay and cocaine determinations

Blood collected into heparin-treated tubes was centrifuged (10 min at 8,000 g) to obtain plasma. Brains were homogenized in 10 volumes of 10 mM sodium phosphate, pH 7.4 with 0.1% Tween-20, and centrifuged as above. Cocaine hydrolase activity in duplicate 50-µl aliquots of plasma or supernatant was assayed by incubating 30 min with ^3^H-cocaine (50 nCi, 18 µM) and measuring liberated ^3^H-benzoic acid after partition into toluene-based fluor for scintillation counting [54]. A related procedure was used to determine levels of ^3^H-cocaine and benzoic acid as previously described [55], in samples prepared in 10^−5^ M DFP to prevent continued enzymatic breakdown. Native rat BChE activity was assayed by a radiometric method [56] using 1 mM ^3^H-acetylcholine as substrate and 10^−5^ M BW284c51 as an acetylcholinesterase inhibitor.

### Viral gene transfer

Experiments used a helper-dependent adenoviral vector that contained cDNA for CocH under regulation by a human ApoE hepatic control region [57], with a bovine growth hormone polyadenylation sequence cloned into a derivative of the p28lacZ hdAd-backbone plasmid. Vector was propagated using the AdNG163 helper virus, as described [58], [59], and particle titers were determined by optical density at 260 nm. Helper virus contamination, determined by plaque assay on HEK-293 cells, was approximately 0.2% for both loaded and empty vectors. Vector delivery was accomplished by rapid injection of a solution containing 10^11^ viral particles through the tail vein in an initial volume of 1 ml following by 0.2 ml of 0.9% sterile NaCl solution.

Prior experience with this viral vector in rats had shown that plasma cocaine hydrolase activity usually rose to a peak in the range of 1000 mU/ml approximately two weeks after injection, with a slow decay over the subsequent weeks and months. This peak level was similar to that attained after purified enzyme (specific activity ∼67,000 mU/mg protein) was injected in a dose of 3 mg/kg. Actual peak magnitudes, however, varied as much as ten-fold from rat to rat for reasons still under investigation. Therefore, plasma cocaine hydrolase activity was routinely determined during the initial transduction phase and weekly during the behavioral experiments. Subjects exhibiting activities less than 150 mU/min at the 2-week sampling point were rejected for further analysis because pilot studies had indicated that such levels were inadequate.

### Statistical Analysis

Beam breaks were taken as an index of locomotor activity and served as the primary dependent measure in this study. Locomotor data averaged across all 3 days of acclimation were compared between groups by analysis of variance (ANOVA). Beam breaks after injections (last 45 min of session) during the training phase (saline and first and last cocaine sessions) and challenge phases (saline and cocaine sessions) were analyzed by 2-way repeated-measures ANOVA with treatment group and condition as factors. The same analysis was used to examine beam breaks during the 45-min habituation periods preceding each injection. After a significant interaction between factors, post-hoc tests were conducted using Fisher’s least significant difference procedure. Total beam breaks after cocaine injections in the 8 training sessions were averaged and compared to antibody and enzyme blood levels by the Pearson Correlation Coefficient. Data collected after the final cocaine challenge were similarly analyzed.
